# Unveiling Carbon Cluster Coating in Graphene CVD on MgO: Combining Machine Learning Force field and DFT Modeling

**DOI:** 10.1021/acsami.4c11398

**Published:** 2024-09-20

**Authors:** Qi Zhao, Hirotomo Nishihara, Rachel Crespo-Otero, Devis Di Tommaso

**Affiliations:** † Department of Chemistry, Queen Mary University of London, London E1 4NS, U.K.; ‡ Institute of Multidisciplinary Research for Advance Materials, Tohoku University, 2-1-1 Katahira, Aoba-ku, Sendai, Miyagi 980-8577, Japan; § Advanced Institute for Materials Research (WPI-AIMR), Tohoku University, 2-1-1 Katahira, Aoba-ku, Sendai, Miyagi 980-8577, Japan; ∥ Department of Chemistry, University College London, London WC1H 0AJ, U.K.; ⊥ Digital Environment Research Institute, Queen Mary University of London, Empire House, London E1 1HH, U.K.

**Keywords:** graphene carbon clusters, MgO, doping, machine learning force fields molecular dynamics, density functional theory

## Abstract

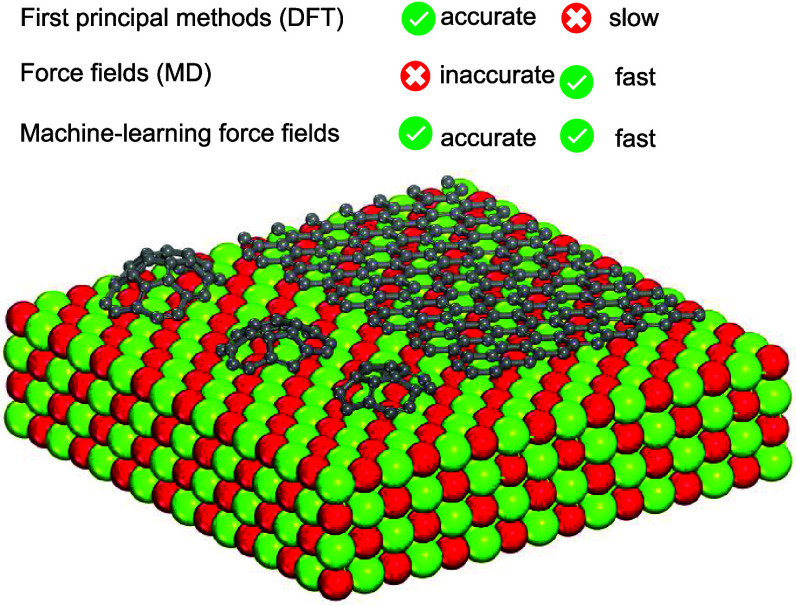

In this study, we investigate the behavior of carbon clusters (C_*n*_, where *n* ranges from 16 to 26) supported on the surface of MgO. We consider the impact of doping with common impurities (such as Si, Mn, Ca, Fe, and Al) that are typically found in ores. Our approach combines density functional theory calculations with machine learning force field molecular dynamics simulations. It is found that the C_21_ cluster, featuring a core–shell structure composed of three pentagons isolated by three hexagons, demonstrates exceptional stability on the MgO surface and behaves as an “enhanced binding agent” on MgO-doped surfaces. The molecular dynamics trajectories reveal that the stable C_21_ coating on the MgO surface exhibits less mobility compared to other sizes C*_n_* clusters and the flexible graphene layer on MgO. Furthermore, this stability persists even at temperatures up to 1100K. The analysis of the electron localization function and potential function of C_*n*_ on MgO reveals the high localization electron density between the central carbon of the C_21_ ring and the MgO surface. This work proposes that the C_21_ island serves as a superstable and less mobile precursor coating on MgO surfaces. This explanation sheds light on the experimental defects observed in graphene products, which can be attributed to the reduced mobility of carbon islands on a substrate that remains frozen and unchanged.

## Introduction

Synthesizing high-quality graphene on a large scale has garnered significant attention for its diverse applications, extending from ultrafast transistors^1^ to transparent and flexible electrodes,^2^ as well as energy storage or conversion devices such as rechargeable batteries,^3^ electric double layer capacitors,^4^ and fuel cells.^5^ Among the several methods for the synthesis of graphene, chemical vapor deposition (CVD) has significant advantages in creating and engineering high-quality graphene thin films.^6^

Historically, transition metals (TM) like Ru,^7,8^ Rh,^9^ Ir,^10,11^ Ni,^12,13^ Pt,^14,15^ Pd,^16^ and Cu.^17,18^ have been used as a substrate in 2D graphene CVD growth. Ongoing efforts have been dedicated to exploring the reaction mechanisms of TM-CVD. A significant experimental discovery has revealed the prevalence of uniform graphene clusters during the initial stages of graphene growth at relatively low temperatures (*T* < 800 K).^19−21^ The aggregation of these clusters at higher temperatures (*T* > 900 K) subsequently initiates the nucleation of graphene. Moreover, Yuan et al. proposed that high-coverage graphene clusters with the feature of less mobility led to the formation of graphene grain boundaries.^22^ The grain boundaries, inherently defective, are expected to degrade the electrical^23,24^ and mechanical^25^ properties of the resulting 2D graphene films. In contrast, for 3D graphene, certain defects can significantly widen its application. In graphene, three types of defects exist: edge sites, topological defects (carbon 5, 7, 8 rings), and basal plane.^26^ Among these defects, their chemical stability (durability) and electrical conductivity exhibit a gradient, with edge sites being the least stable and conductive, followed by topological defects and, finally, the basal plane. In the field of 2D graphene, most researchers focus on basal plane defects and topological defects.^27^ Topological defect is considered as the factor that lowers the performance of 2D graphene.^28^ However, in the field of 3D carbon materials like porous carbons, the majority of defects are edge sites rather than topological defects.^29^ Recently, interesting functions of the topological defects in the 3D graphene material have been revealed. Pirabul et al. revealed that topological defects can anchor metal nanoparticles, leading to better durability compared to edge sites.^30,31^ While it is not possible to disperse metal nanoparticles onto the graphene basal plane, both edge sites and topological defects serve as effective anchoring sites. Yu et al. demonstrated that edge sites and topological defects promote the generation of readily decomposable Li_2_O_2_ during the LiO_2_ battery discharge. This finding is significant because easily decomposable Li_2_O_2_ is a critical factor for high-performing cathodes in Li–O_2_ batteries.^32^ While edge sites are easily decomposed during cycling, topological defects are more stable.^32^ Therefore, for some applications, increasing the number of topological defects in 3D graphene can be beneficial. Understanding the possible causes of forming grain boundaries is crucial to further functionalize graphene-based 3D carbon materials like graphene mesosponge (GMS).

Earth’s abundant oxides (MO_*x*_) including CaO,^33^ SiO_2_,^34^ Al_2_O_3_,^35^ and MgO^29^ have emerged as alternative substrates for the CVD process to grow GMS with a wider range of applications. Compared to graphene grown via TM-CVD, MO_*x*_ substrates offer advantages in controlling the pore structure and crystallinity of the grown 3D graphene, enabling the graphene grown on MO_*x*_ to possess unique properties such as high surface area, developed mesoporosity, and high oxidation resistance,^36,37^ thereby further expanding its utilizations. Nevertheless, a comprehensive understanding of the reaction mechanisms during the CVD process on the MO_*x*_ substrates remains elusive. This includes not only carbon source activation,^29,34,35^ carbon intermediates nucleation,^38^ and the aggregation of carbon units into large-scale graphene^39,40^ but also the notable gap in explaining the experimental observations of grain boundary loops forming in graphene during the CVD process.^29,41−43^ This has been attributed to the reduced mobility of carbon islands on a substrate adopting an orientation that remains frozen and unchanged thereafter.^44,45^

In contrast to other metal oxides that act as solid acid catalysts, MgO is a solid base catalyst that is both active for methane (CH_4_) conversion into GMS and soluble in hydrochloric acid following the formation of GMS.^29^ Our previous experimental-computational work successfully synthesized mesosponge graphene. Using density functional theory (DFT) calculations, we revealed the initial activation of CH_4_ and its conversion to carbon on MgO.^29^ Additionally, the calculated binding energies of *CH_*x*_ (*x* = 0–4) species on various MgO surfaces indicate that *C and *CH exhibit more favorable binding than *CH_2_, *CH_3_, and *CH_4_ (Figure S1 in the Supporting Information), implying that MgO surface possess catalytic sites with the capability to facilitate the activation and dissociation of the carbon source into precursor carbon species.

In this study, a combination of on-the-fly machine learning force fields (ML-FFs) and DFT calculations were used to simulate the early stages of CVD, the behavior of graphene clusters on the MgO substrate. Our focus was on the properties of graphene clusters C_*n*_ (*n* = 16–26) on MgO surfaces. To consider the role of impurities in natural ores used for extracting MgO, the behavior of graphene clusters on MgO doped with common impurities such as Si, Mn, Fe, Ca, and Al was also evaluated.^46^ The results show that the C_21_ cluster is stable and less mobile on the MgO surfaces and that impurities can strengthen the binding of the “magic C_21_” cluster to the substrate, potentially enhancing carbon deposition.

## Methods

### Computational Details

The formation of a graphene cluster in pristine and doped MgO was simulated using DFT and machine learning (ML)-accelerated molecule dynamics (MD). These simulations were conducted with the Vienna Ab initio Simulation Package (VASP) (version 6.4.0).^47,48^ For the DFT calculations, the projector-augmented wave (PAW)^49^ approach was used to describe the electron–ion interaction with a plane-wave energy cutoff of 450 eV. The total energy convergence criterion in the self-consistent field calculation was set to 10^–6^ eV. The exchange–correlation functional was described using the Perdew–Burke–Ernzerhof (PBE)^50^ generalized gradient approximation together with Grimme’s DFT-D3^51^ correction to describe the nonlocal dispersive interactions. The Γ-point sampling was used to sample the Brillouin zone, and the (3 × 3 × 3) *k*-point meshes in the Brillouin zone were used for both MD and static DFT calculations. A 20 Å vacuum layer in the *z*-axis direction was set to avoid self-interaction between periodic images of the unit cell. MD simulations were carried out in the canonical constant-volume, constant-temperature (NVT) ensemble at *T* = 300 K using the deterministic Nose–Hoover thermostat.^52^ The MD simulation was conducted for 10 000 steps with 2 fs time step, covering a total simulation time of 20 ps.

The ML-FF for the MgO system and its interaction with C_21_ were generated using the on-the-fly machine learning method^53^ by conducting MD simulations in the NVT ensemble using a Langevin thermostat to vary the temperatures from 0 to 1100 K. Then, the trained ML-FF was applied on MgO@C_*n*_ (*n* = 16–20 and 22–26), as those models share the same atom interactions. The Bayesian force error for each atom and the root-mean-square errors of predictions with respect to DFT results were used to confirm the accuracy of the ML-FF.^54^ The postprocessing of atomistic data obtained from MD simulations, including the generation of graphical representations of the structures, were done using the OVITO visualization software.^55^ The mean potential distribution in Figure 6 was obtained using optimized structures (C*_n_*/X-MgO) and plotted with the QSTEM^56^ software’s mean potential function. This distribution was generated by averaging the electrostatic potential across a selected plane or direction within the optimized structure.

### Atomistic Models

**Figure 1:**
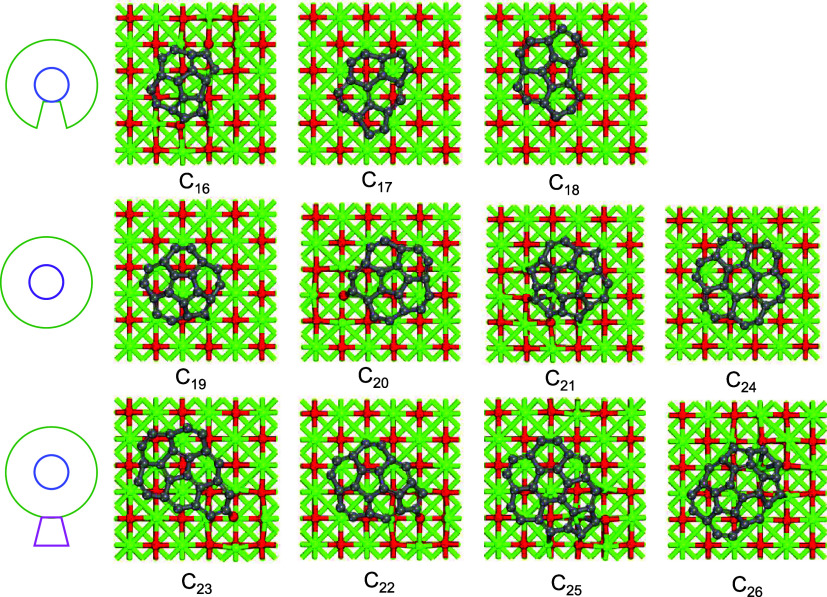
Ground-state structures of the C_*n*_ (*n* = 16–26) clusters on the MgO (100) surface. The models are classified into three groups: C_16_–C_18_ have unclosed core–shell (UCS) structures; C_19_–C_21_ and C_24_ are closed core–shell (CCS) structures; C_22_, C_23_, C_25_, and C_26_ have a core–shell geometry with one or two additional rings (CCS+).

Li et al. used the “on-the-fly” scheme to develop a ML-FF for MgO and investigate the adsorption of water.^53^ In this work, ML-FF was extended to more complex systems, including impurity doping on MgO surfaces. Hereafter, the structural and dynamics of carbon clusters adsorbed on MgO surfaces and impurity doping of MgO surfaces were investigated. Starting from the fully optimized bulk structure of MgO (cubic, *Fm*3*®m*, COD ID: 1000053), a four-layer-thick slab was employed to build the MgO(100) surface, which was then doped with impurities Si, Mn, Fe, Ca, and Al by replacing one surface Mg atom. The supercell of the slab model comprised 2 × 2 repeating unit cells. For each C_*n*_ (*n* = 16–26), the configuration was optimized, and the lowest energy structure was selected as the ground-state structure. The graphene clusters C_20_, C_21_, and C_24_ exhibit a close structural resemblance to corannulene (C_20_H_10_), sumanene (C_21_H_12_), and coronene (C_24_H_12_), respectively. The C_20_, C_21_, and C_24_ were previously speculated to be the dehydrogenated forms of corannulene, sumanene, and coronene, respectively.^57−59^ As exhibited in Figure 1, the structures of C_*n*_ on MgO (labeled as C_*n*_@MgO) can be categorized into three groups: (i) the smaller clusters C_16_, C_17_, and C_18_ exhibited an unclosed core–shell (UCS) geometry; (ii) the medium-sized clusters C_19_, C_20_, and C_21_, and the cluster C_24_ featured a closed core–shell (CCS) structure; (iii) the larger clusters C_22_, C_23_, C_25_, and C_26_ displayed a core–shell structure with one or two additional rings (CCS+). This classification was extended for the C_*n*_ clusters on the X-doped MgO surfaces (X = Si, Mn, Fe, Ca, and Al). The distance between the C_*n*_@MgO clusters in the neighboring cell is listed in Table S3 (Supporting Information).

The initial lattice parameters of graphene were *a* = *b* = 2.460 Å, *c* = 6.800 Å, α = β = 90°, and γ = 120°. To match with the lattice of the slab of MgO (*a* = 8.5 Å, *b* = 8.5 Å, *c* = 18.375 Å, α = γ = β = 90°), the graphene lattice was redefined using the transformation matrix (110/1̅10/001), which gave *a* = 4.920 Å, *b* = 8.522 Å, *c* = 6.800 Å, and α = γ = β = 90°. Then, a (2 × 1) supercell was generated to obtain the graphene lattice parameters *a* = 9.840 Å, *b* = 8.522 Å, *c* = 6.800 Å, and α = γ = β = 90°. Using this procedure, the mismatch along the *a* and *b* directions was δ_*a*_ = (|8.50 – 9.840|/9.840) × 100 = 8.8% and δ*_b_* = (|8.50 – 8.521|/8.521) × 100 = 0.3%, respectively. The overall mismatch rate between graphene and MgO was, therefore,  × 100% = 4.6%, which is lower than 4.7% proposed by Li et al.^60^ Within this mismatch, no buckling or cracking of either the graphene or the MgO substrate was observed after structural relaxation. Using MgO and graphene, a layered structure was built, and then, a periodic structure was generated to obtain a slab model of graphene/MgO with lattice parameters *a* = 8.95 Å, *b* = 8.51 Å, *c* = 36 Å, and α = γ = β = 90°.

## Results and Discussion

### Assessment of the Machine Learning Force Field

**Figure 2:**
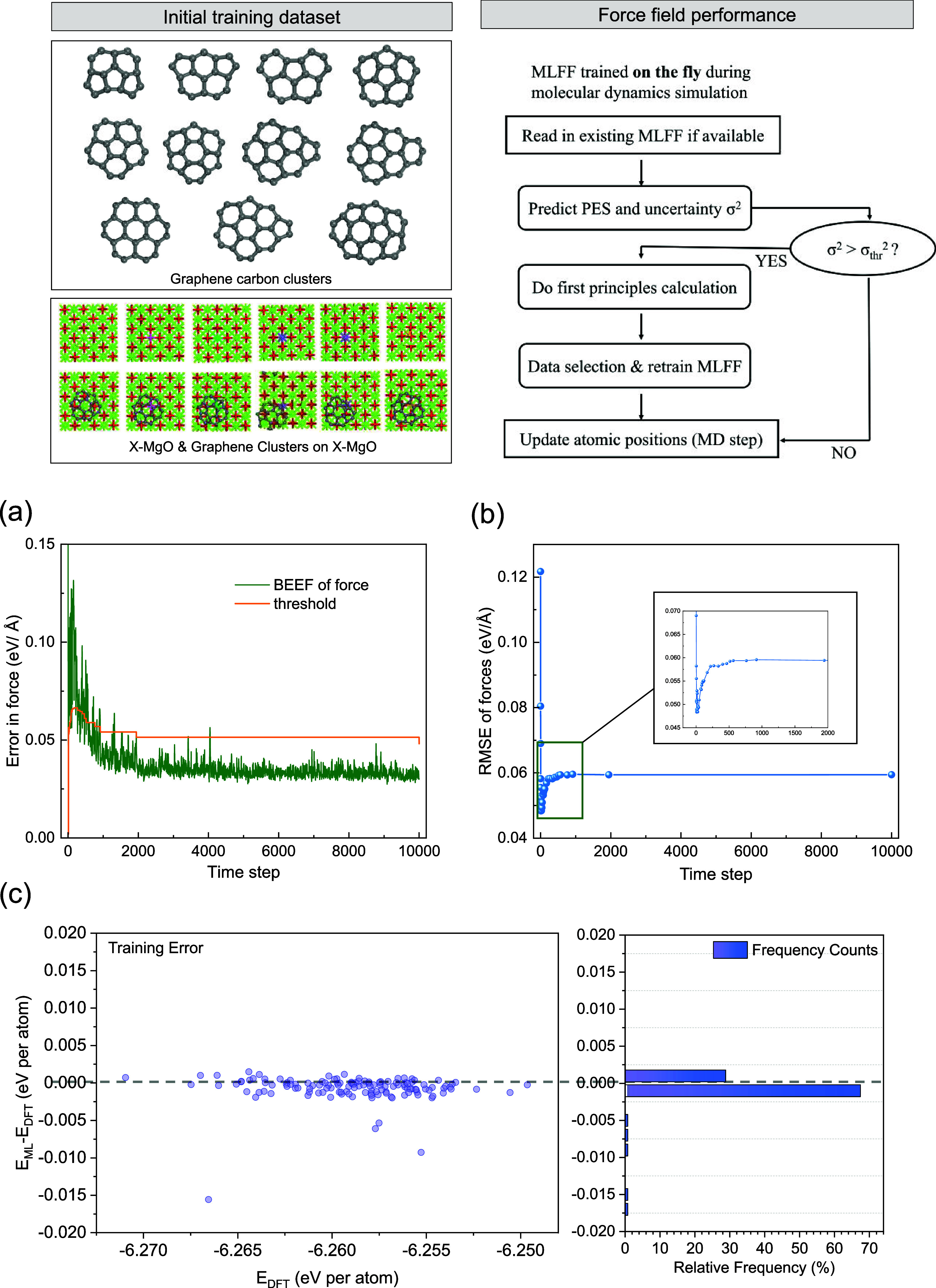
Training and validation of ML-FF obtained for C_21_@MgO. (a) Bayesian error estimation of the force (BEEF) per atom and the threshold criteria set by the on-the-fly ML algorithm in VASP for the generation of the ML-FF. (b) Root-mean-square errors (RMSE) for the predictions of forces with respect to DFT results. (c) Errors of the ML-FF compared to DFT on the evaluation of the energies of 132 randomly selected structures from the ML-FF MD simulation.

As shown in Figure 2, the “on-the-fly” ML-FF generation scheme implemented in VASP follows the following procedure to create a data set for ML-FF training. The ML-FF algorithm estimates the energy, forces, stress tensor, and their uncertainties for a given structure using the existing ML-FF field, which has been widely reported by the Kress group.^47,61−63^ The “on-the-fly” scheme then determines if it should perform a DFT calculation or continue the MD simulation using the current ML-FF. If the predicted uncertainty is too large (σ^2^ > σ_thr_^2^), the energy and forces computed via DFT are added to the data set and used to retrain the ML-FF. Atomic positions and velocities are updated (MD step) using either the force field (if accurate) or the first-principles calculation. If the desired total number of ionic steps is reached, then the process is complete; otherwise, it returns to the initial prediction step.

The “on-the-fly” training simulations were conducted until the uncertainties in the predictions became sufficiently small (as described below). The ML-FF initially developed for bulk MgO was validated by comparing the total energy of the unit cell to the cell volume. Figure S2 demonstrates excellent agreement between the ML-FF and DFT methods. Subsequently, the ML-FF was retrained for the (100), (110), (111), and (310) surfaces. Figure S3 illustrates generally good agreement in energy and geometry features between ML-FF and DFT, particularly for the (100) surface. For the MgO surface doped with X = Fe, Mn, Ca, Al, and Si, the training process was initiated from the ML-FF of pure MgO(100). The accuracy of this ML-FF for the doped MgO system is confirmed in Figure S5 (Supporting Information), which highlights close geometric features between the ML-FF and DFT. Finally, ML-FFs used to simulate C_*n*_@MgO and C_*n*_@X-MgO (where *n* = 16–27) were generated using the C_21_@MgO and C_21_@X-MgO systems, respectively.

The Bayesian error provides an estimate of the out-of-sample error in the context of ML. In VASP, the Bayesian error helps assess the generalizability of the ML-FF generated by using the on-the-fly ML algorithm for evaluating forces and energies. In this work, forces and energy are sufficient to validate the force field because they directly determine the accuracy of atomic trajectories and the stability of the simulated system. In Figure 2, the results from the training and validation of ML-FF obtained for C_21_@MgO are reported. In Figure 2(a), Bayesian error estimations (BEE) for the force and the current threshold criteria are presented. Forces, rather than energy, were considered as a criterion for evaluation of the BEE.^64^ The graph reveals that the error is relatively large in the early stages, gradually diminishing over time to a level below the threshold. Occasionally, BEE experiences sudden spikes, followed by decreases after interference from DFT calculations. After 1000 steps, the Bayesian error mostly remains below the threshold, indicating minimal BEE and achieving the required accuracy in the calculations. Although the threshold undergoes slight changes over time, it generally oscillates around 0.05 eV Å^–1^. In Figure 2(b), the root-mean-square error (RMSE) for the prediction of forces is reported. As the RMSE is calculated only after DFT calculations are performed, it represents the actual errors between the ML-FF and DFT. The RMSE exhibits significant fluctuations in the early stages, which indicates that additional DFT calculations are required. In contrast, the RMSE stabilizes to around 0.06 eV Å^–1^ with longer intervals between data points in the later period, suggesting that ML-FF calculations dominate the MD process, resulting in less frequent updates of the force field. The Bayesian error consistently remained smaller than RMSE, demonstrating the effectiveness of Bayesian inference in capturing errors, even though some errors in the probability model persisted. The accuracy of the ML-FF is confirmed by the small error throughout the simulation.

The generated ML-FF was then validated by considering the energies of a test set, which included 132 structures randomly selected from an MD simulation of C_21_@MgO. For these structures, the DFT energies were also computed, as shown in Figure 2(c). The energy difference between the ML-FF and DFT was at most ±1 meV, and for most structures, the errors were even lower. The comparison in Figures S2–S5 (Supporting Information) of ML-FF and DFT results for the variation of the bulk energy of MgO with the cell volume, the MgO surface energy, and the Mg–O, Mg–Mg, and O–O RDFs further support the accuracy of the ML-FF. In particular, the validation reveals closely matched RDFs, suggesting a very close structure parameter of MgO obtained using ML-FF and DFT. The CPU time required to conduct 20 ps of MD simulations in Table S1 (Supporting Information) also highlights the computational efficiency achieved using the ML-FF compared to DFT. The ML-FF was used to perform the MD simulations and structure optimizations for other carbon cluster sizes C_*n*_@MgO (*n* = 16–26).

The “on-the-fly” method was also used to generate ML-FFs for MgO with the impurities Si, Mn, Fe, Ca, and Al. The BEE for the force per atom and the RMSE for the predictions of forces relative to DFT results are presented in Figure S6. In impurity-doped MgO systems, the threshold may increase to a higher level compared to the pure MgO system. This indicates that the machine learning model identifies complex elements in the new system and retrains the force field accordingly. Although the threshold changes slightly over time, the errors generally decrease to a level below the threshold, typically oscillating around 0.02 eV Å^–1^, which is the force convergence criterion. Furthermore, the RMSE gradually stabilizes to below 0.02 eV Å^–1^ with longer intervals between data points in later stages, ensuring accurate force coverage. In addition, Figure S7 presents the errors of the ML-FF compared to DFT in evaluating the energies of randomly selected structures from the ML-FF MD simulations for X-doped MgO surfaces and the carbon cluster C_21_ on the X-doped MgO surfaces. For systems with impurities, the training errors mostly fall within the range of ±0.025 eV per atom, which is considered an acceptable error.^63,65^

### Formation of Carbon Clusters on MgO: Stability, Mobility, and Structural Characteristics

**Figure 3:**
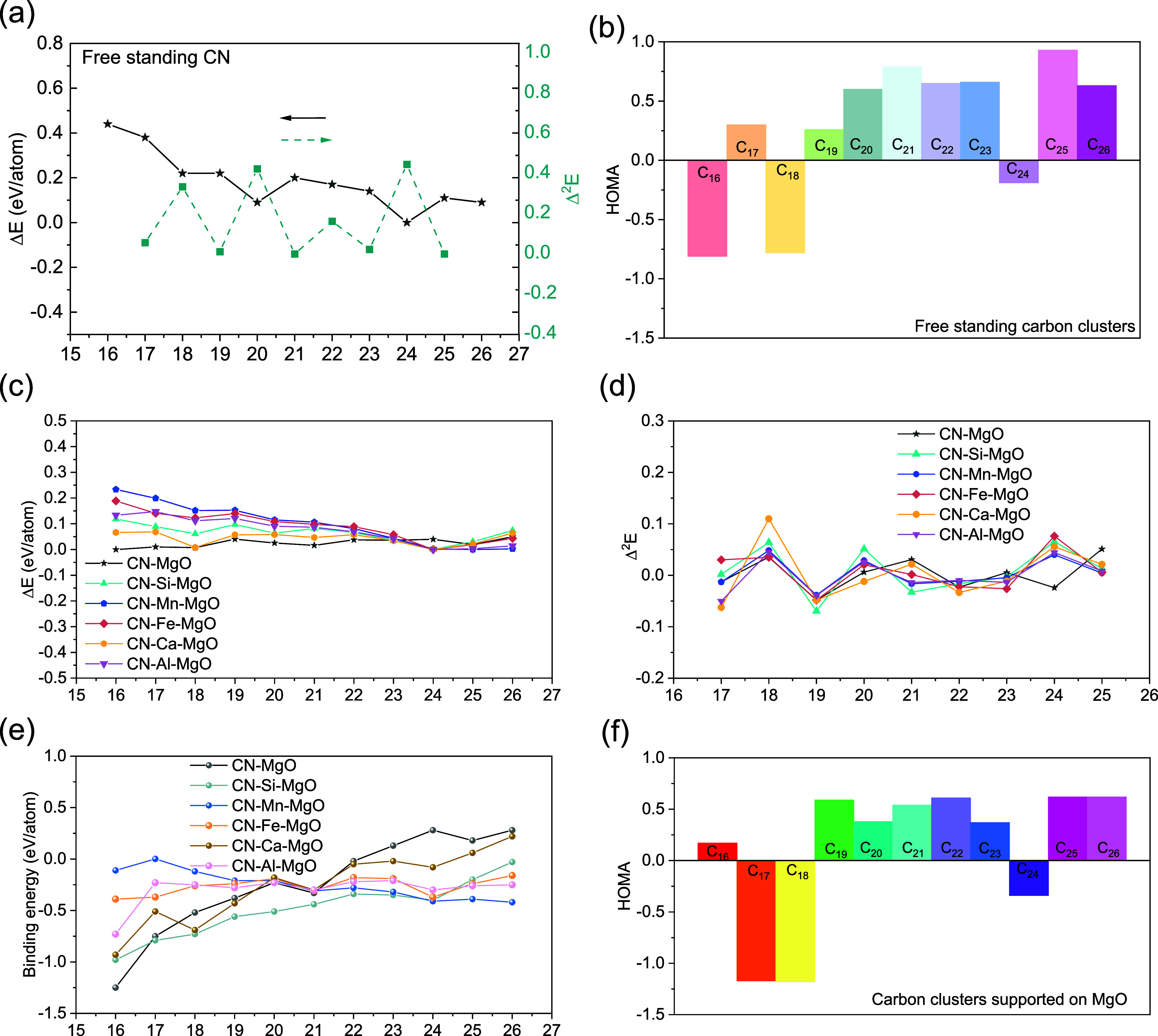
(a) Binding energy (Δ*E*_*n*_) of the free-standing carbon clusters C_*n*_ (*n* = 16–26) and their second derivatives (Δ_2_*E*). (b) Values of the harmonic oscillator model of aromaticity (HOMA) index of the free-standing clusters. (c) Binding energy and (d) second derivatives of C_*n*_@MgO (*n* = 16–26) with and without impurities. (e) Binding energy of C_*n*_ (*n* = 16–26) on X-doped MgO (X = Si, Mn, Fe, Ca, and Al). (f) Values of the HOMA index of C_*n*_@MgO (*n* = 16–26). Static optimization was conducted using the pretrained ML-FF.

Insights into the stability of the free C_*n*_ clusters were obtained from the calculation of the binding energy per atom (Δ*E*_*n*_)^67^
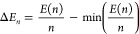
1where the first term is the total energy normalized to the number of atoms (*n*) in the C_*n*_ cluster (for C_*n*_@MgO system, *n* in eq 1 represents the total number of atoms in systems), and the second term is the lowest of the normalized total energies of all systems considered, meaning the “most stable” C_*n*_ cluster is used as the reference for determining the binding energy per atom. The variation of Δ*E*_*n*_ as a function of cluster size is reported in Figure 3(a). Since the variation in binding energy does not always clearly indicate the relative stability of clusters, the second difference in energy (Δ^2^*E*_*n*_) was also employed. This method, previously reported in other works, helps to better illustrate cluster stability^67,68^
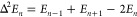
2In Figure 3, a maximum in the Δ^2^*E*_*n*_ profile indicates a more stable cluster compared with its neighboring structures. Following similar studies on bimetallic nanoclusters,^69^ the most stable C_*n*_ structures correspond to the magic-size clusters formed experimentally, and their determination will lead to the growth behavior of these systems. For the stability of the carbon clusters supported on MgO and MgO-doped substrates, the binding energy per atom (*E*_b_) was calculated as
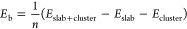
3where the first term is the total energy normalized to the number of atoms (*n*) in the C_*n*_ cluster supported on MgO systems, the second term is the energy of MgO slabs, and the third term is the energy of pure carbon clusters. Comparing the binding energy of free-standing carbon clusters, as shown in Figure 3(a), with the binding energy of carbon clusters supported on MgO, as shown in Figure 3(c), reveals that the binding energy is more negative for C_*n*_@MgO than for free-standing C_*n*_, indicating that more favorably stable configurations are formed on the MgO surface than in gas-phase carbon clusters. Furthermore, by comparing the second difference in energy between gas-phase carbon clusters and C_*n*_@MgO, as depicted in Figure 3(a,d), it becomes evident that Δ^2^*E*_*n*_ is slightly more positive in C_*n*_@MgO. This suggests that the interaction with MgO stabilizes carbon clusters. Consequently, the MgO surface provides effective support and interactions, contributing to the stability of the graphene cluster structure.

The HOMA index for the free-standing and supported carbon clusters, reported in Figure 3(b,f), was used to analyze the effect of binding to the surfaces. HOMA is given by the following equation^70^
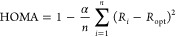
4Here, *R*_*i*_ represents the *i*-th bond length in the analyzed ring, and *R*_opt_ (1.388 Å) represents the reference bond length in a perfect benzene ring. The parameter *n* is the number of carbon–carbon (C–C) bonds within the analyzed ring. Finally, α (257.7 Å^–2^) is a normalization factor that ensures the HOMA index equals 1 for perfectly aromatic benzene and 0 for a hypothetical Kekulé cyclohexatriene ring with a perfect alternation of single and double bonds. The closed core–shell (CCS) structures (C_19_–C_21_, C_24_) and the core–shell geometry with one or two additional rings (CCS+) (C_22_, C_23_, C_25_, and C_26_) show a higher HOMA value in their core ring, indicating the stability of the two types of structures (Figure 3 and Table S2 in the Supporting Information). However, a negative HOMA value was observed on C_24_ (−0.189 for C_24_ and −0.337 for C_24_@MgO). This can be explained by referring to the work of Gao et al., who showed that C_24_ is metastable and forms the most stable C_21_ + 3C structure with three dangling C atoms attached to C_21_.^57^

The binding energies of each carbon atom in these carbon clusters on MgO systems are displayed in Figure 3(e), revealing a general trend of decreasing binding energy with increasing cluster size, except for a notable valley point at the C_21_ position. The electron potential also shows a higher potential on C_21_ compared to other C_*n*_ clusters (Figure S11 in the Supporting Information). This indicates the unique behavior of C_21_ on MgO systems. Moreover, the CCS geometry structure of the C_21_@MgO surface in Figure 1 features a core–shell arrangement with one hexagon in the center surrounded by three pentagons, isolated by three hexagons. Clusters with a CCS geometry generally exhibit higher stability compared to those with UCS or CCS+.^71^

**Figure 4:**
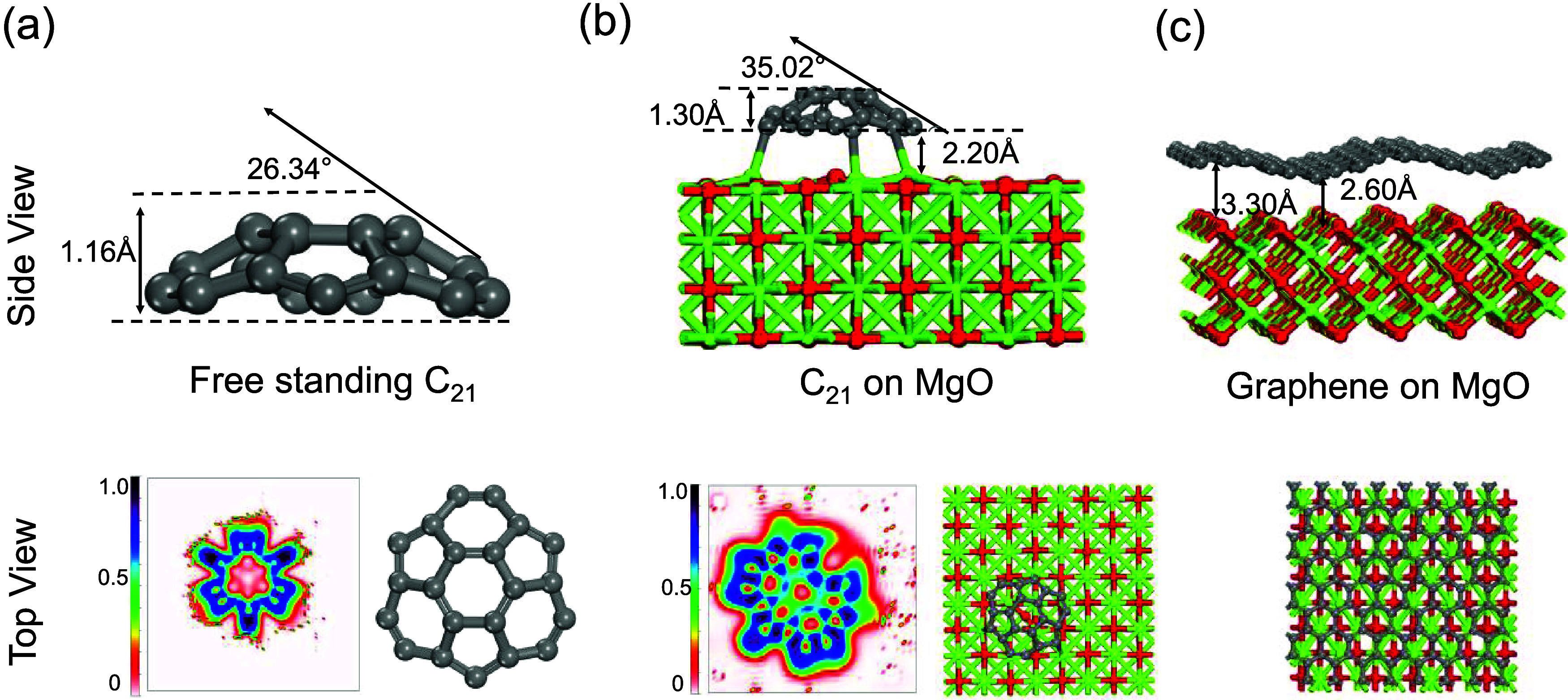
(a) Structural features of free-standing C_21_ and its electron localization function (ELF) and (b) structural features of C_21_@MgO and its ELF. (c) Structural features of graphene@MgO. Static optimization conducted using the pretrained ML-FF.

The geometries of the free-standing C_21_ cluster, C_21_@MgO, and graphene@MgO together with their electron localization function (ELF) are displayed in Figure 4. The values of ELF range from 0 to 1 and indicate the relative electron density in different regions, with higher values signifying stronger interactions.^72^ The comparison of Figure 4(a,b) shows that the carbon cluster C_21_ forms a domelike geometry, which minimizes its edge binding energy.^22,73^ The curvature of the dome-shaped C_21_ cluster effectively maximizes the number of favorable interactions between its edge atoms and the MgO surface. This configuration minimizes the edge formation energy compared to the corresponding planar structure. The C_21_ cluster exhibits a greater tilt angle at the edge when adsorbed onto MgO (35.0°) compared with its free-standing state (26.3°). In addition, the core of C_21_@MgO is less aromatic than free-standing C_21_ due to the lower HOMA (Figure 3 and Table S2). The C_21_ cluster exhibits a stronger interaction with the MgO surface compared to a graphene layer (Figure 4(c)), which is likely due to the shorter distance between C_21_ and the MgO surface (2.2 Å) compared to the distance between the graphene layer and the MgO surface (3.3 Å).

The ELF values around 0.15 (Figure 4(a)) within the central carbon ring (“C_6_ core”) of the free-standing C_21_ cluster reveal weak interactions. This suggests a low electron density within the cluster itself. However, when C_21_ is adsorbed onto MgO, the ELF value in this region significantly increases to 0.55 (Figure 4(b)). This substantial increase in electron density indicates a strong interaction between C_21_ and the MgO support. Furthermore, Figure 4(b) shows a more localized “C_6_ core” in C_21_@MgO, indicating a more distorted toroid-shaped region around it. This distorted volume is composed of three pentagons isolated by three hexagon regions with a highly concentrated electron density, as evidenced by the ELF value of 0.85.

**Figure 5:**
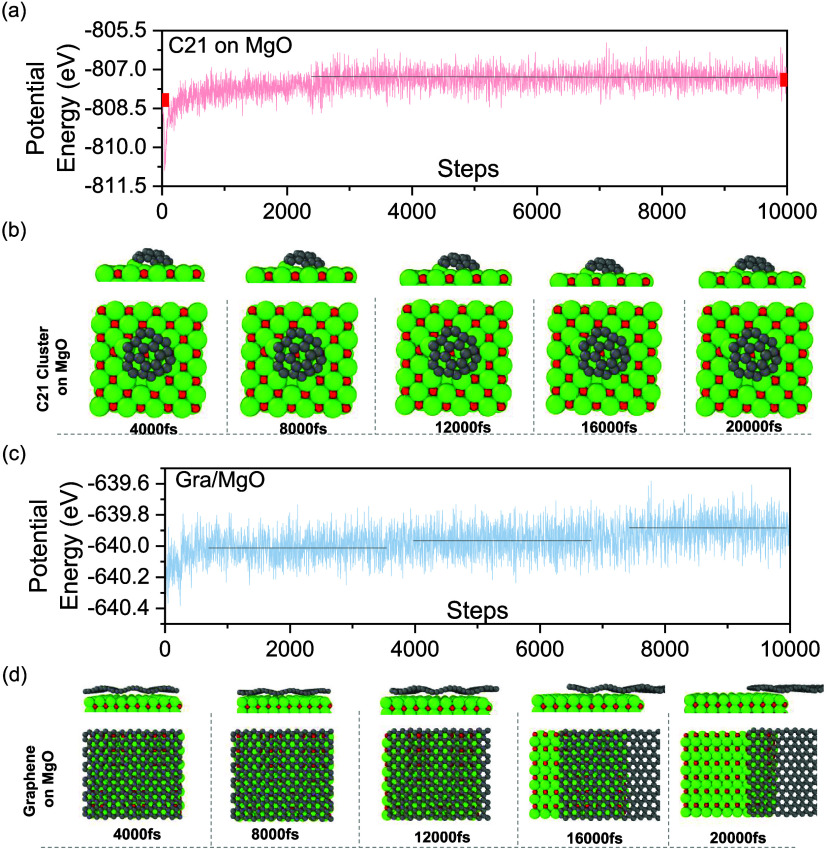
Variation of the potential energy with time for (a) C_21_@MgO and (c) graphene on MgO (Gra@MgO). Snapshots from ML-FF MD simulations of (b) C_21_@MgO and (d) Gra@MgO visualized using OVITO.

MD simulations of C_21_@MgO and graphene@MgO revealed that the C_21_ cluster exhibited not only stability but also low mobility. For C_21_@MgO, the potential energy remained stable after 20 000 fs during the MD simulation (Figure 5(a)) because C_21_ remained attached to the same surface sites throughout the simulation (Figure 5(b)). Even during the heating process from 1 to 1100 K, C_21_ still remains stable with low mobility, as depicted in Figure S9. In contrast, the potential energy of graphene@MgO exhibits fluctuations over time (Figure 5(d)). This behavior arises from the flexibility of graphene, allowing it to move across the MgO surface in a wave-like motion between 4000 and 8000 fs (Figure 5(d)). By 16 000 fs, the entire graphene layer has moved significantly away from its initial position on the MgO surface, as evidenced by the substantial fluctuations of the potential energy (Figure 5(c)). These observations support other computational findings by Jiao et al., who demonstrated the high mobility of graphene on the growth substrate.^74^ The other carbon clusters either exhibit the flexibility and mobility of graphene on the MgO surface or instability, with carbon bonds breaking within rings (Figure S9 in the Supporting Information).

### Effect of MgO Doping on the Formation of Magic C_21_ Clusters

**Figure 6:**
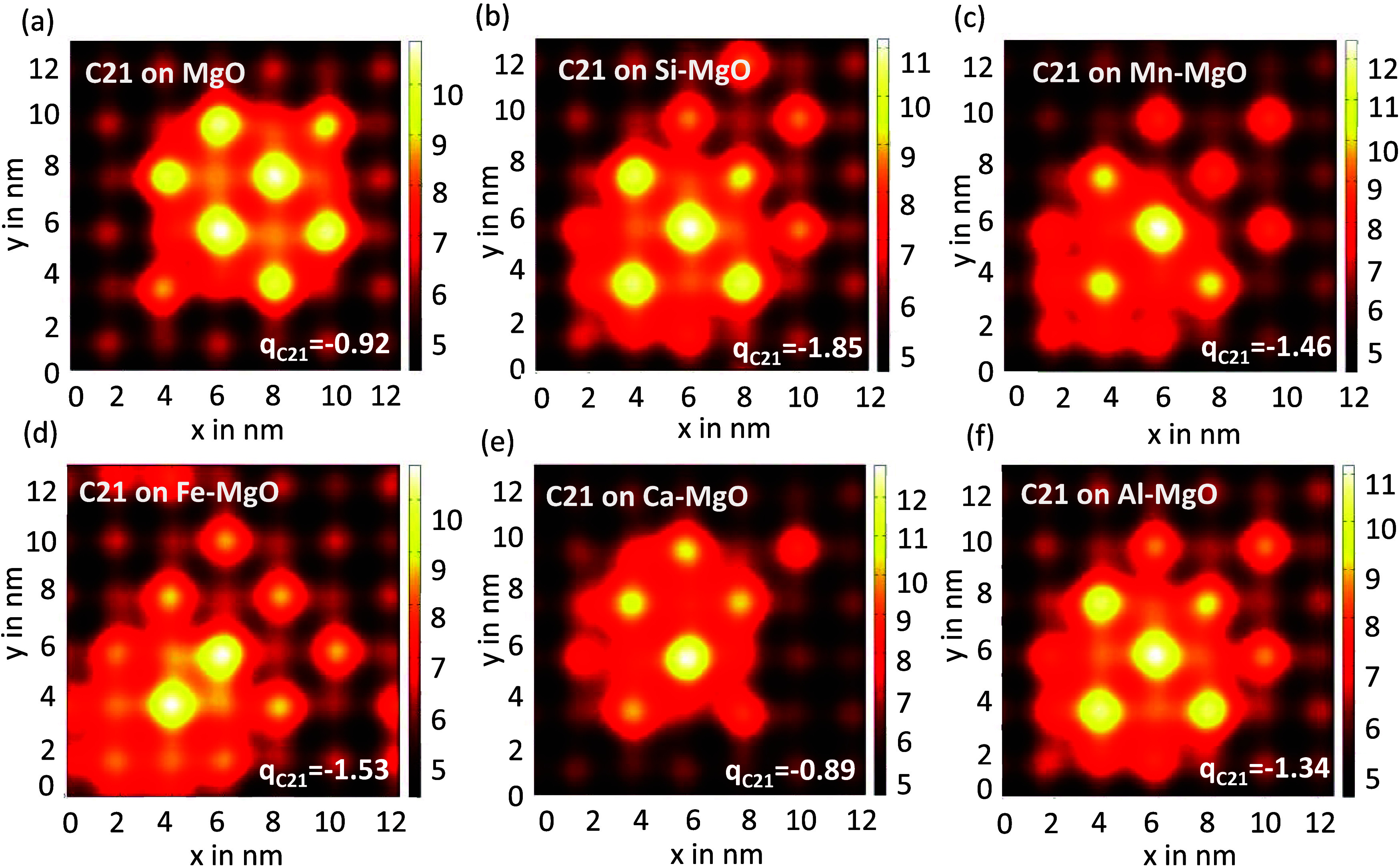
Mean potential of the C_21_ cluster on (a) MgO and MgO doped with (b) Si, (c) Mn, (d) Fe, (e) Ca, and (f) Al. The C_21_ Bader charge obtained between C_21_ and X-MgO (X = Mg, Si, Mn, Fe, and Ca) systems are labeled at the right bottom.

The introduction of Si on the MgO surface, as illustrated in Figure S4(b), results in a markedly enhanced binding energy between the surface and the C_21_ cluster, which translates to a characteristically low surface mobility exhibited by C_21_. To understand the mechanism behind this strong binding, the density of states (DOS), shown in Figure S8, reveals a significant decrease in the band gap (from 1.8 to 1.0 eV) for Si-doped MgO compared to pure MgO, which favors the adsorption of species on the surface.^75^Figure 5 shows the mean potential distribution for a C_21_ cluster adsorbed on the MgO surface. Regions with higher mean potential values likely correspond to areas with greater electron density. This suggests stronger electron localization, which, in turn, implies enhanced interactions and binding forces, contributing to the structural stability of C_21_ on the MgO surface. On the other hand, the areas of low mean potential arise from weaker interactions, allowing for increased flexibility or structural alterations in these regions. The lower potential may also suggest a more diffused electron cloud, hinting at charge transfer, as shown in the Bader charge value presented in Figure 6 with significantly high charge transfer, *q* = −1.85, between C_21_ and Si-MgO compared to other systems. The negative Bader charge value obtained for C_21_ indicates a charge transfer from the MgO surface to C_21_ (Figure 6). Moreover, the central region of C_21_ is characterized by a higher potential on the MgO-doped surfaces, especially with Si, Mn, Ca, and Al, which are the normal impurities that exist in ores,^46^ compared to pure MgO surface. The structures of MgO doped with these elements are displayed in Figure S10. The results confirm that the presence of impurities could enhance the likelihood of carbon island or cluster deposition on the template surface. This, in turn, may control graphene grains as the high concentration of nuclei or graphene islands/clusters on the MgO surface is inevitably associated with the formation of graphene grain boundaries.

## Conclusions

This investigation focused on the behavior of carbon clusters C_*n*_ (*n* = 16–26) on MgO and MgO-doped (Si, Mn, Fe, Ca, and Al) surfaces using an integrated ML-FF and DFT computational methodology. Considering formation energies, second derivatives of binding energy, and electron potential results, C_21_ is identified as the cluster with a higher stability. This is attributed to its closed core–shell structure with three hexagons isolated by three pentagons. Analysis of the trajectory of the ML-FF MD simulations conducted at different temperatures reveals the less mobile feature of C_21_ on MgO surfaces compared to flexible graphene and other size C_*n*_ clusters. The impact of common impurities (Si, Mn, Fe, Ca, and Al) found in natural ores on the behavior of the “magic C_21_” cluster on MgO surfaces was also considered. Binding energy and electron potential analyses show that impurities enhance the binding of C_21_ on MgO surfaces. The formation of “magic C_21_” clusters on MgO surfaces could induce reduced mobility of carbon islands on a substrate that remains immobile, potentially leading to growth defects. This work provides a possible insight into the puzzling experimental observations related to grain boundary formation during the chemical vapor deposition of graphene.

## Supporting Information Available

Binding energies of CH_*x*_ (*x* = 0–4) on different types MgO surface (Figure S1); comparison of machine learning force field (ML-FF) and DFT calculations of MgO bulk: total energy against the cell volume (Figure S2); comparison of ML-FF and DFT calculations of the MgO surfaces: (a) surface energies; (b–d) radial distribution function, *g*(*r*), of different MgO surfaces (Figure S3); comparison of ML-FF and DFT calculations of C_*n*_@MgO (*n* = 16–26): (a) optimized energy of free-standing C_*n*_ clusters; (b) C_21_ on MgO doped with Si, Al, Ca, Fe, and Mn (Figure S4); comparison of the radial distribution function, *g*(*r*), obtained from MD simulations using DFT and ML-FF of (a) pure MgO and MgO doped with (b) Si, (c) Mn, (d) Al, (e) Ca, and (f) Fe (Figure S5); Bayesian error estimation of the force per atom and the threshold criterions set by the on-the-fly ML algorithm in VASP for the generation of the ML-FF for: (a) pure MgO; MgO doped with (c) Fe, (e) Al, (g) Mn, (i) Ca, and (k) Si; the root-mean-square error (RMSE) for the predictions of forces with respect to DFT results for: (b) pure MgO; MgO doped with (d) Fe, (f) Al, (h) Mn, (j) Ca, and (l) Si (Figure S6); errors of the ML-FF compared to DFT on the evaluation of the energies of randomly selected structures from the ML-FF MD simulations for X-doped MgO surfaces and the carbon cluster C_21_ on the X-doped MgO surfaces (X = Si, Mn, Fe, Ca, and Al) (Figure S7); total density of state distribution and band gap of (a) MgO and MgO doped with (b) Si (c) Mn (d) Fe (e) Ca, and (f) Al (Figure S8); snapshots of the ML-FF-MD simulations of C_*n*_ (*n* = 16–26) on the MgO (100) surface for temperatures ranging from 1 to 1100 K; green ball, red ball, and gray ball represent Mg, O, and C, respectively (Figure S9); atomistic models of C_*n*_ (*n* = 16–26) on the MgO(100) surface doped with Si, Mn, Fe, Ca, and Al (Figure S10); mean potential of C_*n*_ (*n* = 16–27) clusters on the MgO (100) surface; values in eV (Figure S11); comparison of CPU time necessary to conduct 20 ps of MD simulations using ML-FF and DFT; values in s (Table S1); the C–C distance and HOMA value in the core of carbon cluster (Table S2); and the distance (Å) between the C*_n_* clusters in neigboring cell (Table S3) (PDF)
