# Supplementary material for: Unveiling Carbon Cluster Coating in Graphene CVD on MgO: Combining Machine Learning Force field and DFT Modeling
Source: ACS Appl Mater Interfaces. 2024 Sep 20;16(39):53231–41. doi: 10.1021/acsami.4c11398 (PMC11450684; doi:10.1021/acsami.4c11398)
Supplement: Supplementary file 1 — am4c11398_si_001.pdf [file am4c11398_si_001.pdf]

Supporting Information

Unveiling Carbon Cluster Coating in Graphene CVD on MgO: Combining Machine Learning Forcefield and DFT Modelling

Qi Zhao [a], Hirotomo Nishihara [b, c], Rachel Crespo-Otero [d*], Devis Di Tommaso [a,e*]

[a] Department of Chemistry, School of Physical and Chemical Sciences, Queen Mary University of London, London, E1 4NS, UK

[b] Institute of Multidisciplinary Research for Advance Materials, Tohoku University, 2-1-1 Katahira, Aoba-ku, Sendai, Miyagi, 980-8577, Japan.

[c] Advanced Institute for Materials Research (WPI-AIMR), Tohoku University, 2-1-1 Katahira, Aoba-ku, Sendai, Miyagi, 980-8577, Japan.

[d] Department of Chemistry, University College London, London, WC1H 0AJ, UK

[e] Digital Environment Research Institute, Queen Mary University of London, Empire House, London E1 1HH, UK

*Corresponding authors.

E-mail addresses: hirotomo.nishihara.b1@tohoku.ac.jp (H. Nishihara); r.crespo-otero@ucl.ac.uk (R. Crespo-Otero), d.ditommaso@qmul.ac.uk (D. Di Tommaso)

Table of Contents

S1. Binding energies of CH_x (x = 0–4) on MgO 2

S2. Assessment of machine learning forcefields 3

S3. MgO doped with Si, Mn, Al, Ca, and Fe 7

S4. ML-FF MD simulations of CVD growth 9

S5. MgO doped with Si, Mn, Al, Ca, and Fe 11

S1. Binding energies of CH_x (x = 0–4) on MgO

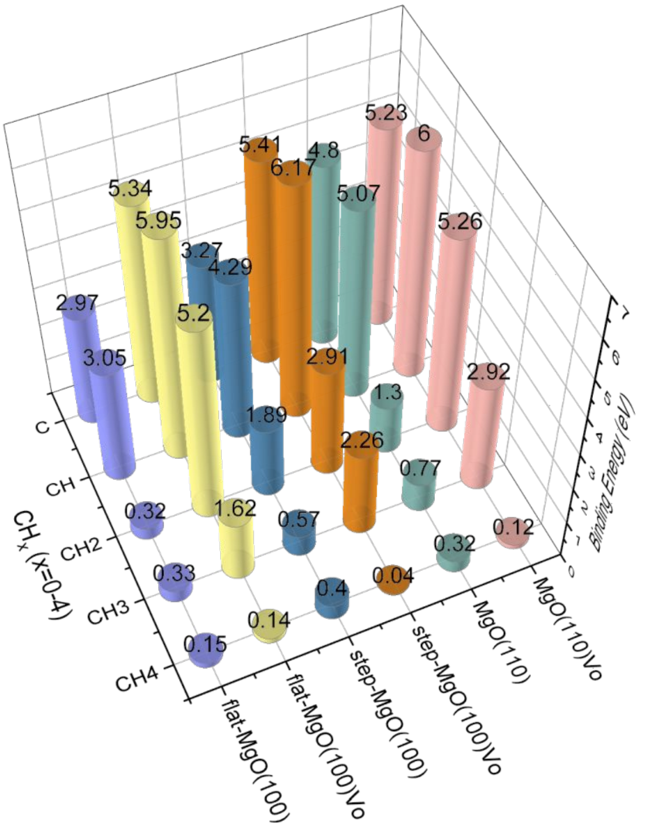

Figure. S1. Binding energies of CH_x (x = 0–4) on different types MgO surface.

S2. Assessment of machine learning forcefields

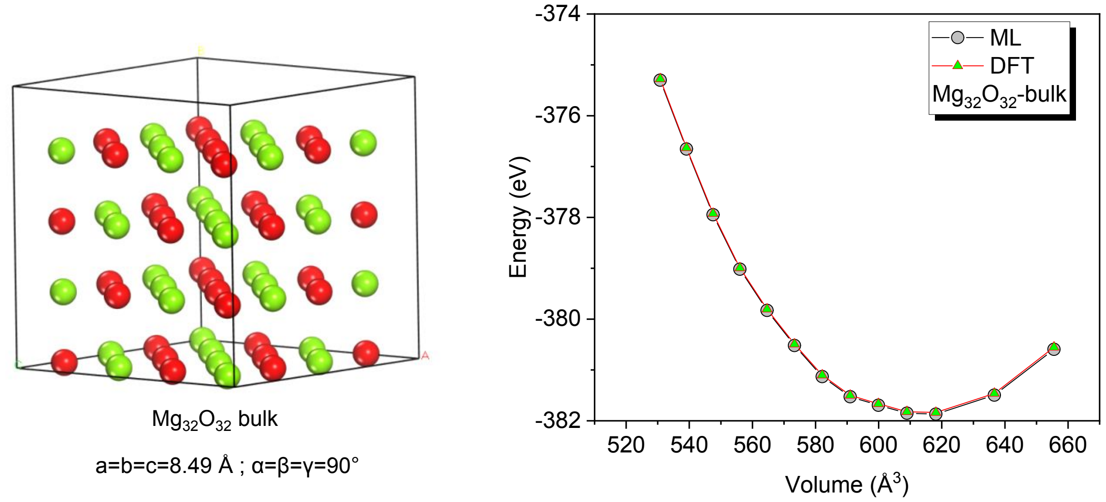

Figure. S2. Comparison of machine learning forcefield (ML-FF) and DFT calculations of MgO bulk: total energy against the cell volume.

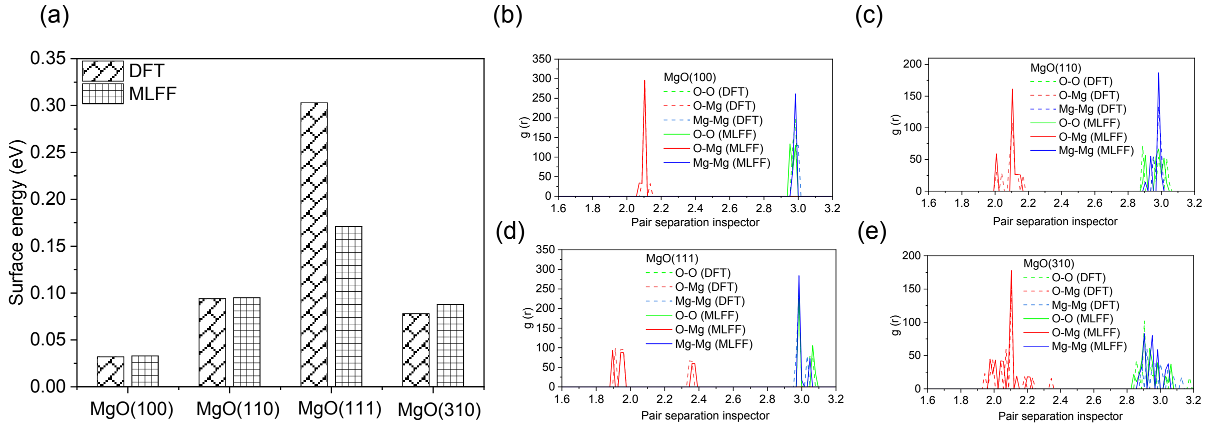

Figure. S3. Comparison of ML-FF and DFT calculations of the MgO surfaces: **(a)** Surface energies; **(b)-(e)** Radial distribution function, g(r), of different MgO surfaces.

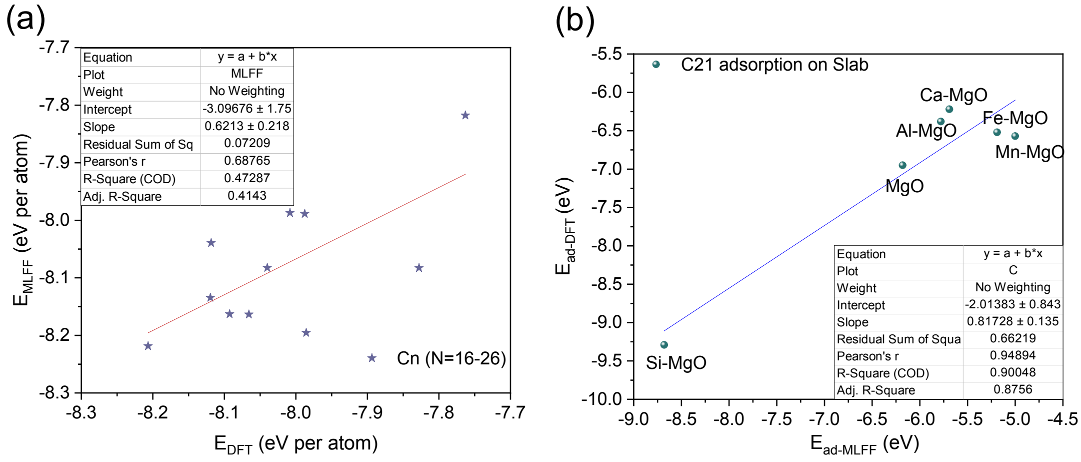

Figure. S4. Comparison of ML-FF and DFT calculations of C_n@MgO (n = 16-26): **(a)** Optimised energy of free-standing C_n clusters. **(b)** C_{21} on MgO doped with Si, Al, Ca, Fe, Mn.

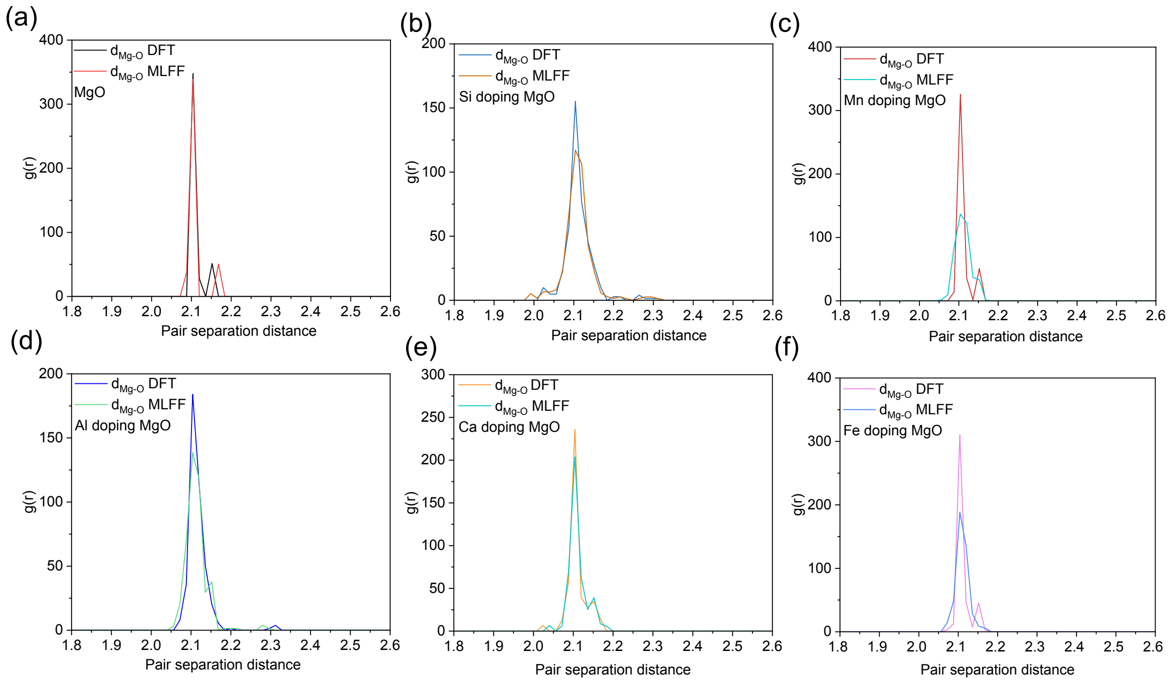

Figure. S5. Comparison of the radial distribution function, g(r), obtained from MD simulations using DFT and ML-FF of **(a)** pure MgO and MgO doped with **(b)** Si, **(c)** Mn, **(d)** Al, **(e)** Ca, and **(f)** Fe.

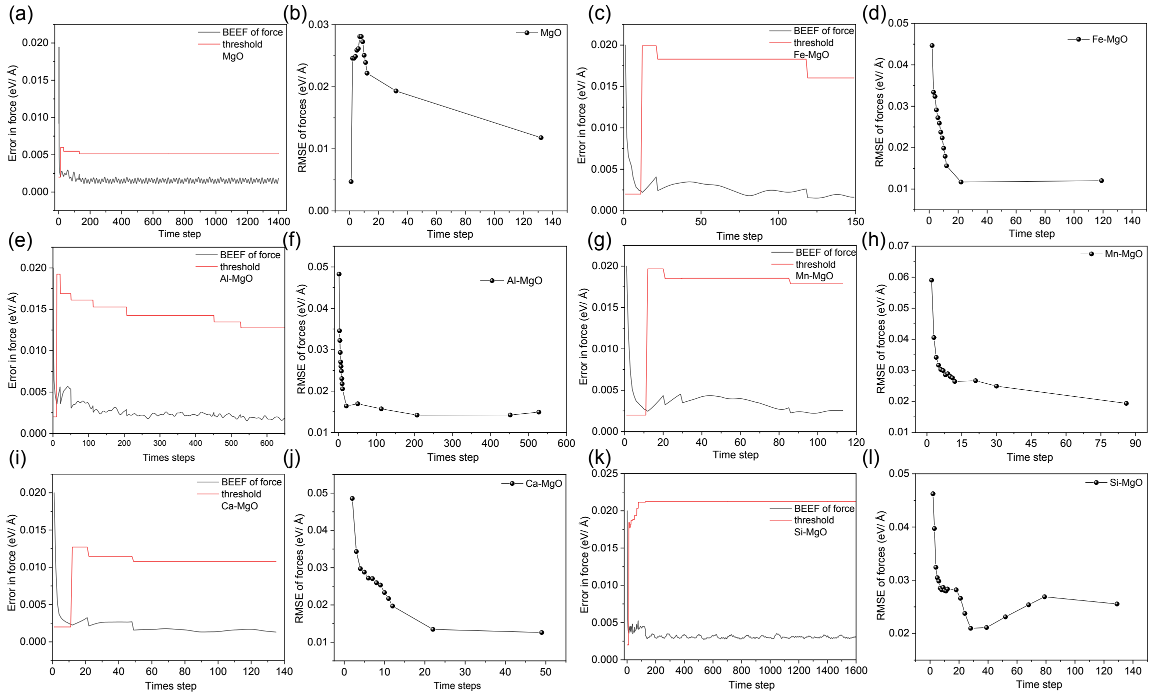

Figure S6. Bayesian error estimation of the force per atom and the threshold criterions set by the *on-the-fly* ML algorithm in VASP for the generation of the ML-FF for: **(a)** pure MgO; MgO doped with **(c)** Fe, **(e)** Al, **(g)** Mn, **(i)** Ca, and **(k)** Si. The root-mean-square errors (RMSE) for the predictions of forces with respect to DFT results for: **(b)** pure MgO; MgO doped with **(d)** Fe, **(f)** Al, **(h)** Mn, **(j)** Ca, and **(l)** Si.

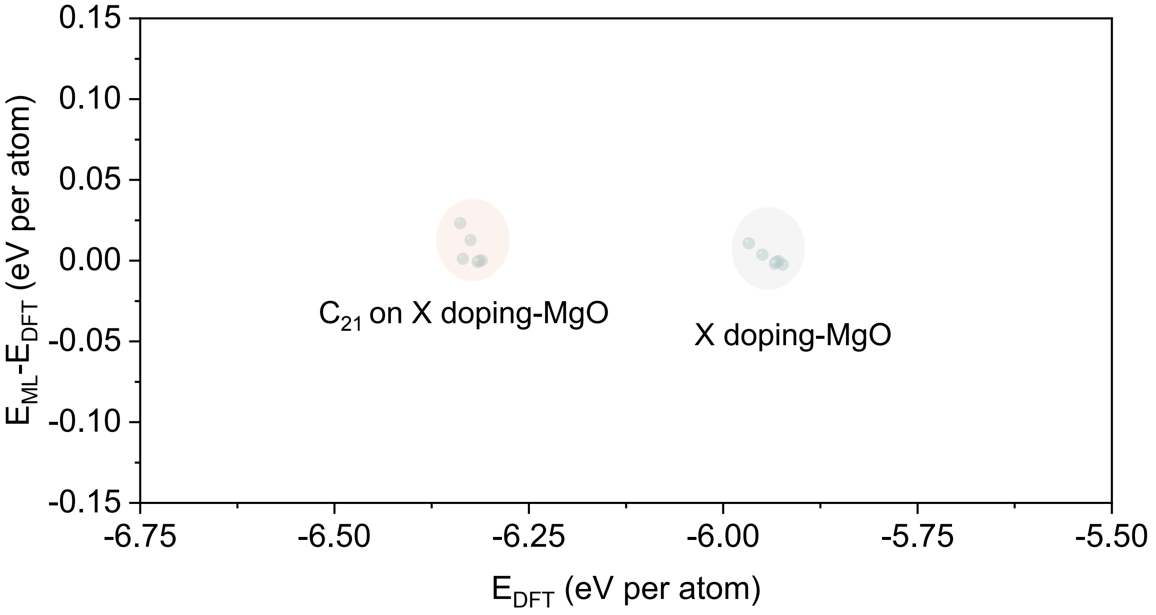

Figure S7. Errors of the ML-FF compared to DFT on the evaluation of the energies of randomly selected structures from the ML-FF MD simulations for X-doped MgO surfaces and the carbon cluster C_{21} on the X-doped MgO surfaces (X = Si, Mn, Fe, Ca and Al).

Table S1. Comparation of CPU time necessary to conduct 20 ps of MD simulations using ML-FF and DFT. Values in s.

	DFT (MD) (total CPU time)	ML-FF (training) (total CPU time)	ML-FF (MD) (total CPU time)
$Mg_{32}O_{32}$(100)	1140.099	166.497	2.347
$Mg_{64}O_{64}$(110)	30362.354	267.531	2.304
$Mg_{48}O_{48}$(111)	19549.164	143.328	2.527
$Mg_{48}O_{48}$(310)	12757.356	160.516	2.646
$Si_1Mg_{53}O_{54}$	67452.289	9462.119	16.074
$Mn_1Mg_{53}O_{54}$	61424.266	7209.707	3.892
$Fe_1Mg_{53}O_{54}$	78514.930	6165.746	3.982
$Ca_1Mg_{53}O_{54}$	71419.180	5202.110	3.886
$Al_1Mg_{53}O_{54}$	78390.898	13123.066	4.187

Table S2. The C-C distance and HOMA value in the core of carbon cluster

	d_{C-C} (Å)						HOMA
C_{16}							
C_{16}@MgO	1.416	1.470	1.460	1.463	1.418		0.010
C_{17}	1.466	1.400	1.465	1.341	1.347		0.304
C_{17}@MgO	1.469	1.424	1.393	1.391	1.530	1.460	-1.166
C_{18}	1.449	1.571	1.448	1.405	1.404		-1.131
C_{18}@MgO	1.459	1.471	1.551	1.473	1.459		-1.616
C_{19}	1.427	1.481	1.428	1.440	1.441		0.109
C_{19}@MgO	1.435	1.420	1.440	1.425	1.435		0.510
C_{20}	1.431	1.431	1.431	1.431	1.431		0.519
C_{20}@MgO	1.439	1.445	1.448	1.426	1.447		0.259

C_{21}	1.377	1.427	1.379	1.429	1.379	1.427	0.785
C_{21}@MgO	1.394	1.429	1.340	1.420	1.362	1.459	0.538
C_{22}	1.432	1.416	1.415	1.433	1.440		0.579
C_{22}@MgO	1.420	1.437	1.429	1.433	1.433		0.528
C_{23}	1.438	1.407	1.407	1.438	1.434		0.596
C_{23}@MgO	1.466	1.431	1.437	1.437	1.431		0.248
C_{24}	1.455	1.457	1.456	1.456	1.455	1.456	-0.186
C_{24}@MgO	1.458	1.460	1.463	1.459	1.457	1.463	-0.337
C_{25}	1.360	1.378	1.410	1.405	1.387	1.398	0.925
C_{25}@MgO	1.396	1.398	1.380	1.424	1.443	1.453	0.623
C_{26}	1.440	1.376	1.441	1.375	1.440	1.397	0.630
C_{26}@MgO	1.442	1.385	1.443	1.385	1.441	1.392	0.623

Table S3 The distance (Å) between the C_n clusters in neigboring cell

	Distance along X direction	Distance along Y direction
C_{16}@MgO	9.108	7.659
C_{17}@MgO	8.151	6.284
C_{18}@MgO	7.634	5.697
C_{19}@MgO	6.586	6.907
C_{20}@MgO	6.798	6.180
C_{21}@MgO	6.935	6.441
C_{22}@MgO	6.175	6.826
C_{23}@MgO	5.593	6.054
C_{24}@MgO	5.239	5.259
C_{25}@MgO	5.783	5.438
C_{26}@MgO	5.502	5.505

S3. MgO doped with Si, Mn, Al, Ca, and Fe

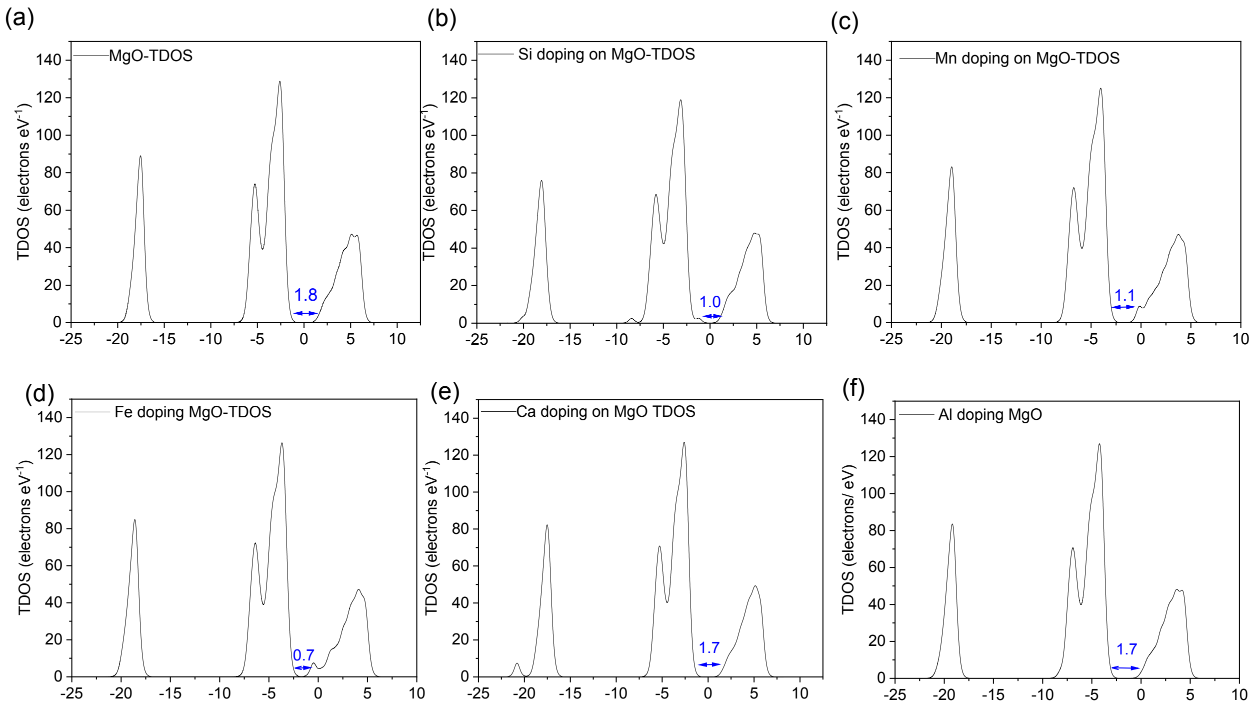

Figure S8. Total density of state distribution and band gap of **(a)** MgO, and MgO doped with **(b)** Si **(c)** Mn **(d)** Fe **(e)** Ca, and **(f)** Al.

S4. ML-FF MD simulations of CVD growth

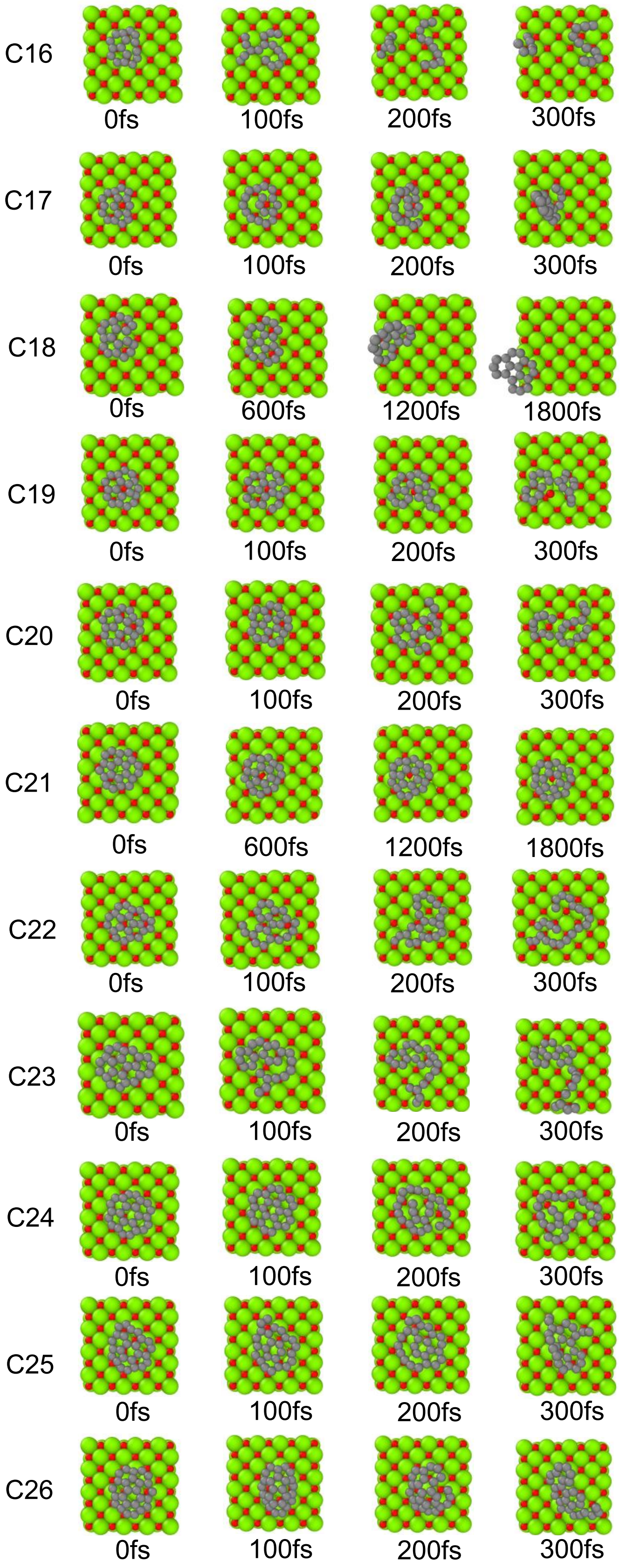

Figure. S9. Snapshots of the MLFF-MD simulations of C_n (n = 16–26) on the MgO (100) surface for temperatures ranging from 1K to 1100 K. Green ball, red ball and gray ball represent Mg, O and C, respectively.

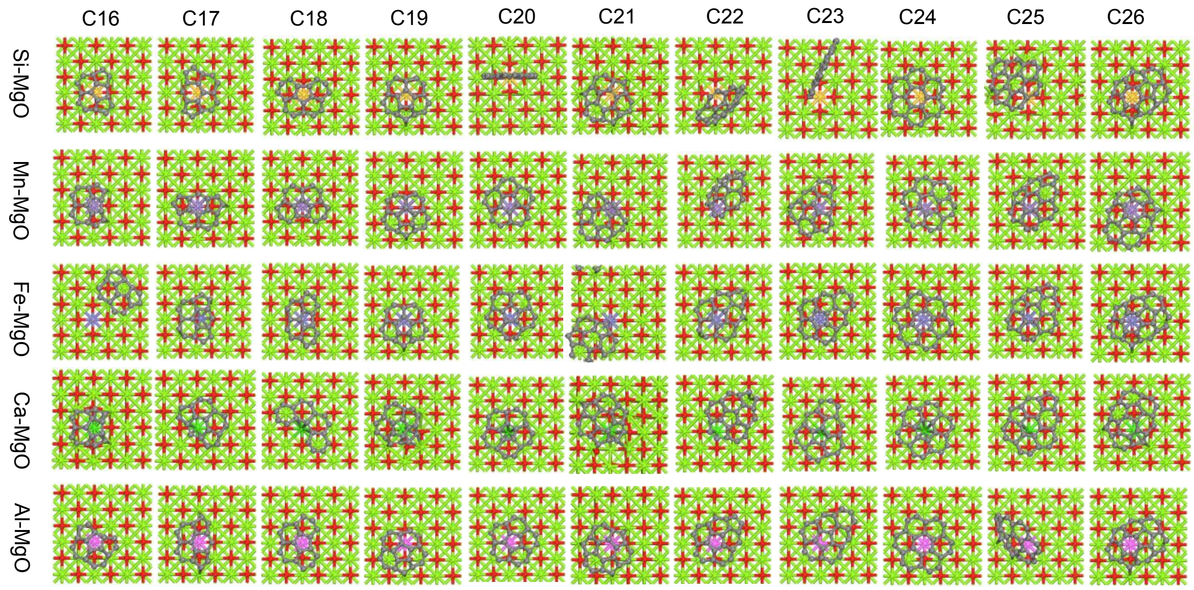

Figure. S10. Atomistic models of C_n (n = 16–26) on the MgO(100) surface doped with Si, Mn, Fe, Ca, and Al .

S5. MgO doped with Si, Mn, Al, Ca, and Fe

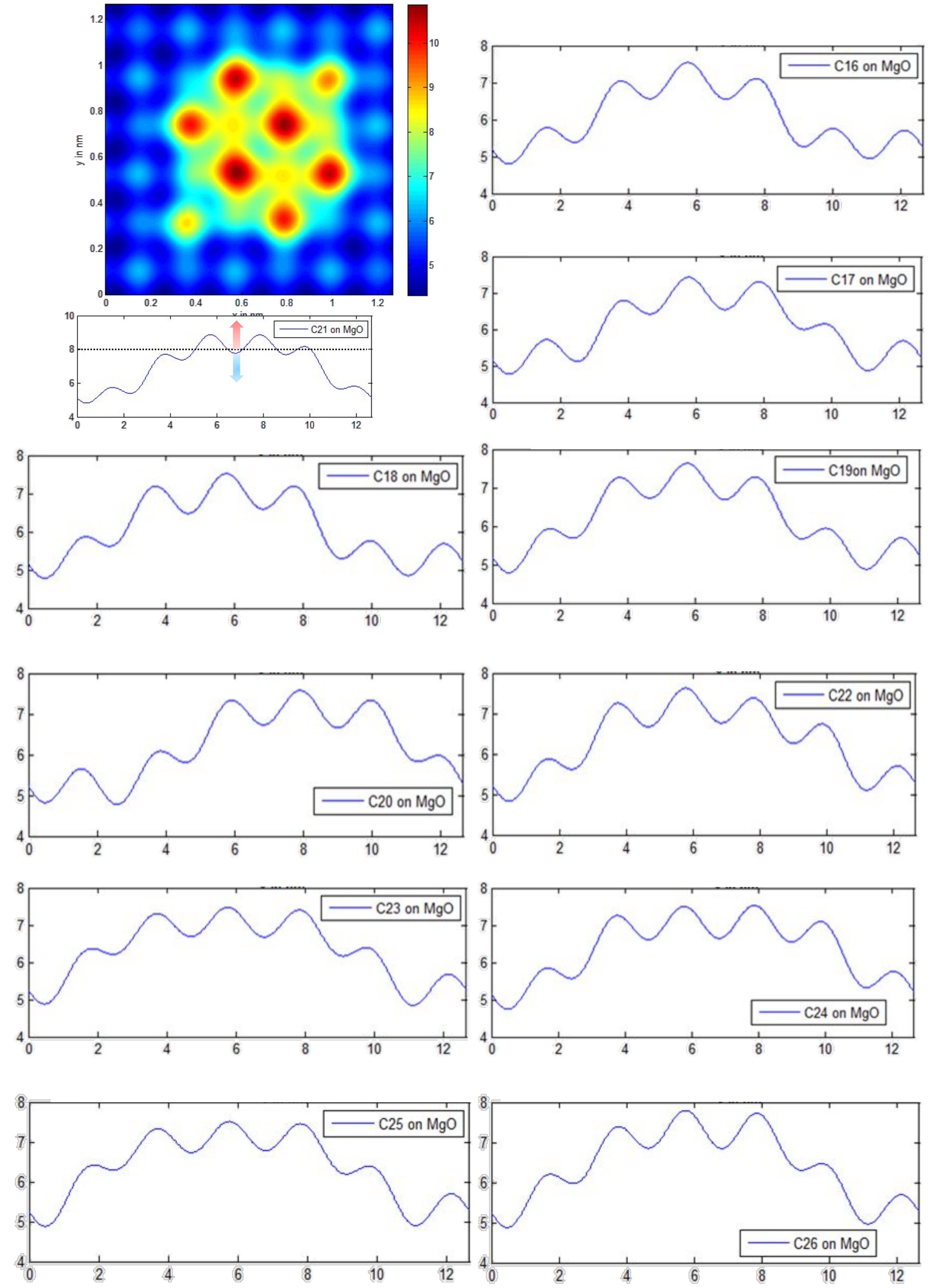

Figure S11. Mean potential of C_n (n=16-27) clusters on the MgO (100) surface. Values in eV.
